# Primary splenic hydatidosis in a 25-year-old man: a case report

**DOI:** 10.4076/1757-1626-2-8017

**Published:** 2009-08-25

**Authors:** Amitesh Aggarwal, Vishal Sharma

**Affiliations:** Department of Medicine, University College of Medical SciencesDelhi 110001India

## Abstract

Hydatid disease is common in sheep rearing areas. It can involve many organs especially liver and lung. Isolated involvement of spleen is rare. We present the case of 25-year-old male presenting with heaviness in left upper abdomen and found to have isolated splenic hyditadosis.

## Case presentation

**Figure 1.**
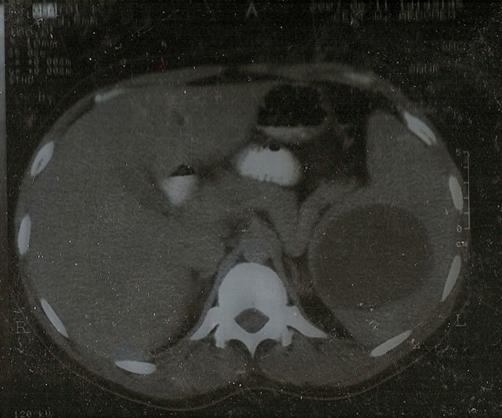
CT abdomen showing splenic hypointense homogenous lesion.

A 25-year-old male, resident of Noida (Uttar Pradesh), presented with history of sensation of heaviness of left upper abdomen since three months. The sensation was not related to meals, exercise or any other activity and was present continuously. There was no history of dyspepsia, prolonged fever or weight loss, abdominal pain, altered bowel habits or urinary complaints. There was no other significant medical history. The examination was unremarkable except for presence of a palpable tip of spleen. His routine investigations revealed a Hb-12.3 gm%, TLC-9700/cu mm, DLC of N80L18M1E1, and an ESR of 54 mm at end of first hour. Ultrasonography of abdomen revealed an enlarged spleen (15.7 cm) with well encapsulated cystic lesion measuring 7.9 cm × 6.3 cm × 7.8 cm. The characteristic double line sign was seen. The Contrast enhanced CT revealed an enlarged spleen (16 cm) with a well defined homogenous hypodense lesion with an enhancing capsule seen in posterior part of spleen measuring 8.4 × 7.2 cm in size (Figure 1). The liver and other abdominal organs and chest were normal. ELISA for Echinococcus was positive. A diagnosis of primary hydatid cyst of spleen was made and patient was put on oral albendazole for a month and advised splenectomy which the patient refused.

## Discussion

Hydatid disease is common in areas where sheep and cattle rearing are important. It most commonly affects liver and lung. Primary and sole involvement of spleen is rare. In a review of around 900 patients of abdominal hydatidosis only 16 were due to isolated splenic hydatidosis [1]. In a series from India of 183 patients of abdominal hydatidosis only 4 had primary splenic hydatidosis [2]. The diagnosis is usually a result of workup for left upper abdominal pain or purely incidental. Painful cysts are usually due to a larger size.

The diagnosis is suspected when a cystic lesion is seen on abdominal sonography. Differentials of a cystic lesion in the spleen include dermoid cysts, epidermoid cysts, cystic hemangiomas or lymphangiomas, abscess or hematoma [1,3]. Eosinophilia and a positive serology in form of a positive ELISA or indirect hemagglutination favour the diagnosis. Radiological techniques can be useful to diagnose hydatid cyst. Cystic lesion with a fluid layer of different density due to hydatid sand indicates hydatidosis. The presence of daughter cysts within a larger cyst or cysts in other organs is most pathognomic [3]. In view of the life threatening complications surgical management should be offered to all the patients. While small peripheral lesions can be managed with conservative spleen saving procedures like partial cystectomy and omentopexy, splenectomy remains the management of choice in most patients [1,4]. Preoperative albendazole therapy is believed to reduce the risk of intraoperative rupture. Puncture, aspiration, injection of scolicidal and reaspiration (PAIR) as utilized in hepatic lesions is not recommended in splenic disease although some recent reports indicate favourable results with this approach [5]. All in all primary splenic hydatidosis is a rare entity which must be considered in the differential of a cystic lesion of spleen in endemic areas. In view of potentially life threatening complications, surgery should be offered to the patient.

## Abbreviations

DLC: differential leucocyte count
Hb: haemogloblin
TLC: total leukocyte count
